# Effects of Different Maturity Stages on Antioxidant Content of Ivorian Gnagnan (*Solanum indicum* L.) Berries 

**DOI:** 10.3390/molecules15107125

**Published:** 2010-10-15

**Authors:** Denis N’Dri, Luca Calani, Teresa Mazzeo, Francesca Scazzina, Massimiliano Rinaldi, Daniele Del Rio, Nicoletta Pellegrini, Furio Brighenti

**Affiliations:** 1 Department of Public Health, University of Parma, via Volturno 39, 43125 Parma, Italy; E-Mails: ndri_denis@yahoo.fr (D.N.); luca.calani@nemo.unipr.it (L.C.); mazfer@libero.it (T.M.); francesca.scazzina@unipr.it (F.S.); daniele.delrio@unipr.it (D.D.); furio.brighenti@unipr.it (F.B.); 2 Department of Industrial Engineering, University of Parma, viale GP Usberti 181/A, 43124 Parma, Italy; E-Mail: massimiliano.rinaldi@unipr.it (M.R.)

**Keywords:** *Solanum indicum* L., phytochemical compounds, total antioxidant capacity, colour, maturity stage

## Abstract

Gnagnan (*Solanum indicum* L.) is a spontaneous plant widely distributed in Ivory Coast. During ripening stages, *Solanum indicum* L. presents different colours (green, yellow and red) and is reported to contain several albeit poorly characterized antioxidant compounds. This paper describes in detail the antioxidant profile (ascorbic acid, carotenoids and polyphenols), antioxidant capacity (FRAP test and Folin-Ciocalteau assay) and the colour changes of Gnagnan berries at different ripening levels. Ascorbic acid content was similar in green and yellow berries, but significantly lower in red ones. Red berries showed a higher content of carotenoids compared to green and yellow ones. Regarding polyphenols, several phenolic acids and flavonoids were found in all berries. The content of caffeoylquinic acids, caffeic acid, flavonol glycosides and naringenin was higher in red berries, while the content of *p*-coumaric acid and feruloylquinic acids was similar among the three colours. The FRAP assay increased with the ripening process, whereas total polyphenols were similar among berries. Significant differences were found for the colorimetric indexes among products of different degrees of ripening. The present results show the important role of the ripening stage in increasing the antioxidant content of Gnagnan berries.

## 1. Introduction

*Solanum indicum *L., also known as “African nightshade” or “bitter berries”, is cultivated in many parts of Africa and the Arabian Peninsula for culinary purposes. In Ivory Coast *Solanum indicum *L., known locally as “Gnagnan”, is consumed fresh or sun dried, usually made as a soup alone or mixed with other vegetables. Like tomato fruits, Gnagnan assumes different colours, from green to yellow, and finally red during its ripening period. During the harvest period, from July to October, the local populations eat the vegetable frequently because the product is fresh and savoury [1], though little attention is paid to the degree of ripeness. After this period, the dried berries become available on the markets, but the fruits are not particularly appreciated because they become tasteless. 

Traditionally *Solanum indicum *L. is also used as an herbal remedy for several diseases such as diarrhoea, malaria and prostate diseases. Despite these supposedly beneficial effects on human health, few scientific studies focused on *Solanum indicum *L. to assess its composition and demonstrate its health effects. In an *in vivo *study, Bahgat *et al.* [2] showed that a standardised extract of *Solanum indicum* L. containing more than 0.15% of chlorogenic acids prevented the development of hypertension in rats. Three studies on edible plants from Iran and India demonstrated that these berries had the highest content in phenolics compared to the other plants analysed [3,4,5]. Considering the scarce information available on the composition of Gnagnan, the purpose of this study was to characterize the antioxidant content of the berries at different stages of maturation. This characterisation should allow the definition of the optimal ripening degree at which this food should be consumed in order to introduce the highest amount of antioxidant phytochemicals. 

## 2. Results and Discussion

### 2.1. Colour analysis

**Figure 1:**
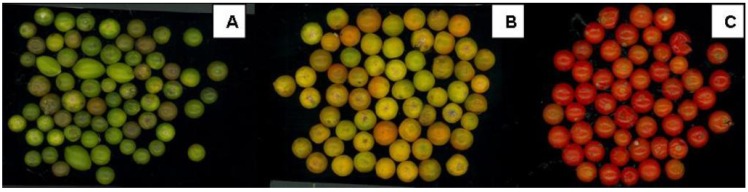
*Solanum indicum *L. berries at different ripening stages (green, A; yellow, B; red, C).

Images of the three ripening stages of *Solanum indicum* L. berries considered in this study are shown in Figure 1; the corresponding colorimetric indexes are presented in Table 1.

**Table 1:** Colour indices of green, yellow and red *Solanum indicum* L. berries.

Colourimeter^a^			
	L*	a*	b*
**Green**	44.1 ± 5.5b	-5.1 ± 3.4c	26.4 ± 8.4b
**Yellow**	48.8 ± 3.8a	28.5 ± 3.9b	40.9 ± 6.4a
**Red**	42.9 ± 2.2b	33.7 ± 3.5a	30.7 ± 30.6b
**Image analysis^a^**			
**Green**	51.0 ± 6.3a	-20.1 ± 2.5b	51.8 ± 7.9ab
**Yellow**	49.5 ± 2.2ab	31.8 ± 8.9a	57.6 ± 2.1a
**Red**	40.1 ± 3.7b	41.0 ± 8.7a	44.7 ± 3.9b

^a^ Values are expressed in colorimetric units and presented as mean ± SD (n = 10). Means in columns for each berry followed by different letters differed significantly (p ≤ 0.05).

When berries are on the trees they take 6-7 days and 1-2 days for turning from stage A to B and from stage B to C, respectively. On the contrary, once the berries are picked, ripening times become shorter: 2-3 days from A to B and 1 day from B to C. For this reason, a nutritional evaluation of the different ripening stages is needed in order to make recommendations.

Significant differences among products of different ripening degree (A, B and C) were found for their colorimetric indexes. In particular, a* index, as previously reported for tomato berries, changed from negative (green colour) to positive (red colour) as a consequence of both chlorophyll degradation and lycopene synthesis [6]. Both data, from colorimeter and image analysis, discriminate ripening levels: in particular, image analysis could represent a cheap method for evaluating *Solanum indicum* L. ripening level. 

### 2.2. Ascorbic acid

Ascorbic acid content of *Solanum indicum* L. at different ripening degrees is reported in Table 2. The ascorbic acid content was similar in green and yellow berries, but lower in red berries. Comparing the present data with other works on *Solanum* genus plants, tomatoes showed higher concentrations of ascorbic acid with respect to all *Solanum indicum* L. berries analysed. For instance, ripe fruits of 12 tomatoes for fresh consumption and 15 processing cultivars had an average ascorbic acid content of 17 mg/100 g [7], whereas cherry tomatoes grown in cold greenhouses and harvested at different times of the year showed a reduced ascorbic acid content from 16 to 44 mg/100 g [8]**.**

**Table 2:** Ascorbic acid, α-carotene, β-carotene and lycopene content of *Solanum indicum* L. at different maturation stages.

	Ascorbic acid	α-carotene*	β-carotene	lycopene
**Green**	8.46 ± 0.81a	N.Q.	0.02 ± 0.01a	N.D.
**Yellow**	8.54 ± 0.28a	N.Q.	0.06 ± 0.06a	N.D.
**Red**	6.67 ± 0.32b	0.15 ± 0.06	1.16 ± 0.34b	1.84 ± 0.52

Mean values ± SD (n = 3) of mg/100 g of fresh weight. Means in columns for each berry followed by different letters differed significantly (p ≤ 0.05). N.Q. not quantifiable; N.D. not detected; * quantification as β-carotene equivalents.

Regarding the change of ascorbic acid content during ripening, the reduction observed in *Solanum indicum* L. was in agreement with previous data [9],where the maximum content was estimated in tomatoes that turned yellow in colour whereas advanced ripening caused a decrease in ascorbic acid content. This trend was likely due to the oxidative degradation of ascorbic acid, since the ripening cell absorbs high amounts of oxygen as a result of increasing rate of cell respiration, this representing the characteristic physiological change in climacteric fruits and vegetables at ripeness [9]. 

### 2.3. Carotenoids

**Figure 2:**
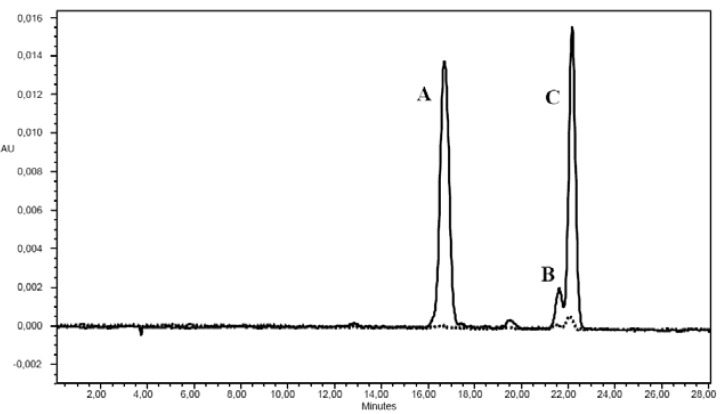
Chromatogram of carotenoids in red (^___^) and yellow (----) *Solanum indicum *L. berries. A: lycopene; B: α-carotene; C: β-carotene.

Carotenoids increased during ripening (Table 2). The concentration of carotenoids was much lower in green and yellow berries, in which lycopene was not detected and α-carotene was not quantifiable (Figure 2), than in red berries. As already demonstrated in tomato [10], the carotenoid content is involved in the colour change, reaching the maximum concentration when the fruit becomes bright red*.* Among carotenoids detected in berries of red colour, lycopene showed the highest concentration, as previously reported for tomato fruits in which lycopene is the most abundant carotenoid, representing approximately between 80 and 90% of total pigments [10,11]. However, *Solanum indicum* L. at full ripeness showed a lycopene concentration at the bottom of the range shown for tomatoes, whose lycopene concentration has been reported to be highly variable ranging from 1.86 to 14.62 mg/100 g of fresh weight (FW) [12]. Conversely, the amount of β-carotene in full ripe *Solanum indicum* L. was higher than that reported in different tomato full ripe fruits [10,11].

Regarding β-carotene, its concentration was about 1.2 mg/100 g in the fully ripe berries, increasing about 60 and 20 times with respect to green and yellow berries, respectively. β-Carotene is a pro-vitamin A and it can be converted into retinol in the intestine and other tissues [13]. Retinol is essential for general growth, visual function and embryonic development, as well as in epithelial tissues differentiation. In humans, the Recommended Dietary Allowance (RDA) of vitamin A is 900 and 700 μg/day (expressed as retinol equivalents) for male and female adults, respectively, corresponding to 5,400 μg/day and 4,200 μg/day of β-carotene equivalents [14]. Considering the β-carotene content of *Solanum indicum* L., 100 g of product are able to warrant almost 30% of RDA. As the vitamin A deficiency is still a public health problem in the sub-Saharan Africa [15], the consumption of these berries represents a useful measure to prevent chronic deficiency of this vitamin. Moreover, this fruit is traditionally consumed cooked, and cooking procedures improve the nutritional value of S*olanaceae* as either β-carotene and lycopene bioavailability is higher in cooked or processed tomatoes than in raw ones [16,17]. 

### 2.4. TAC and Total Polyphenols

In Table 3, TAC values measured by FRAP assay and the total polyphenols measured by Folin-Ciocalteu assay are reported. TAC values increased linearly with ripening, whereas total polyphenols were slightly higher in the yellow berries. Few works evaluated the total polyphenols of *Solanum indicum *L., probably because this vegetable is not consumed in the Western countries. Only three recent works have quantified phenolic compounds in some wild edible plants from India and Iran, including *Solanum indicum* L., finding 700 mg of total phenolics per 100 g of dry matter [3,4,5]. Considering that the average moisture content of *Solanum indicum* L. was approximately 80% (data not shown), the content of total polyphenols per 100 g on dry weight basis was almost 710 mg/100 g for yellow berries, in agreement with previous data [3,4,5]. Moreover, the content of total phenolics is in agreement with previous data obtained in tomato, as Lenucci et al. [18] reported a total phenolic content ranging from 97 mg to 137 mg/100 g of fresh weight for cherry tomatoes.

**Table 3:** Total antioxidant capacity measured by FRAP and total phenol content of *Solanum indicum* L. at different maturation stages.

	FRAP _*_	Total polyphenols _**_
**Green**	0.58 ± 0.06a	102.09 ± 22.64a
**Yellow**	0.63 ± 0.05a	135.15 ± 47.04a
**Red**	0.88 ± 0.11b	123.48 ± 34.38a

Data expressed as mean values ± SD (n = 3);* mmol Fe(II)/100 g of fresh weight; ** mg catechin equivalents/100 g of fresh weight. Means in columns for each berry followed by different letters differed significantly (p ≤ 0.05).

Regarding TAC values, red berries showed a value of almost 0.9 mmol of FRAP per 100 g of fresh weight, higher than that previously reported for tomatoes. In fact, a recent study reported that the FRAP value ranged from 0.34 mmol/100 g to 0.62 mmol/100 g for cherry tomatoes [19] and Pellegrini *et al.* [20] reported a FRAP value equal to 0.51 mmol/100 g for salad tomatoes. Such differences can be justified by the lower moisture content of *Solanum indicum *L. with respect to that of tomatoes that ranges from 93 to 94% [10,21]. Furthermore, the round shape of *Solanum indicum* L. and its size (about 1 cm of diameter) increase the ratio between surface (skin) and fruit total weight, possibly explaining the higher TAC values with respect to tomato fruits [21]. 

It must also be pointed out that food matrixes are complex and antioxidant compounds may be present in different forms, hydrophilic and lipophilic, free, bound to other macromolecules as well as physically entrapped in cellular structure. Therefore they can be partly insoluble in specific solvents used during conventional extraction [22]. In *Solanum indicum* L., as reported for tomato [22], carotenoids are probably present in crystalline form deposited in chromoplasts and flavonoids are probably concentrated in the peel where they are physically entrapped in the pectin network. Therefore, as both antioxidant compounds are partly soluble in the solvents used for the TAC measurements, a slight underestimation of the FRAP value of the berries analysed cannot be excluded. 

### 2.5. Phenolic compounds

**Figure 3:**
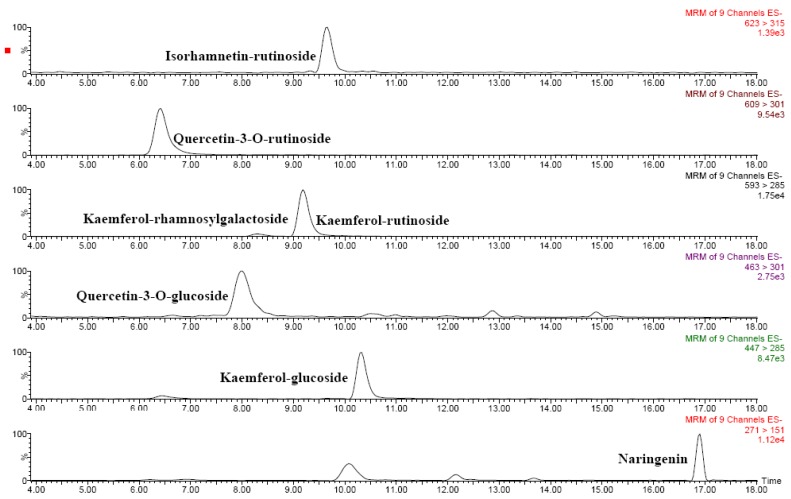
HPLC-ESI-MS/MS chromatograms of main flavonoids in red *Solanum indicum *L..

**Figure 4:**
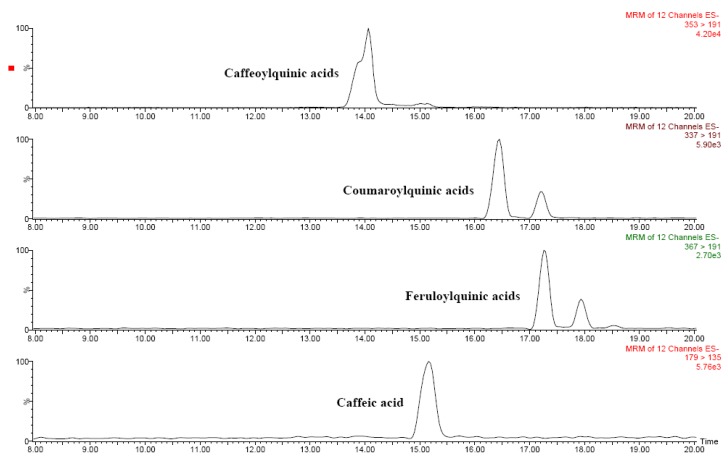
HPLC-ESI-MS/MS chromatograms of main phenolic acids in red *Solanum indicum *L..

Several phenolic acids as well as several flavonoids were identified in *Solanum indicum* L. berries by means of liquid chromatography–tandem mass spectrometry (Table 4). Figure 3 and Figure 4 show the chromatograms of the main flavonoids and phenolic acids, respectively. Chlorogenic and hydroxycinnamic acids were the main phenolic acids, whereas several *O*-glycosylated flavonols and naringenin were quantified among flavonoids. Based on these results, *Solanum indicum* L. has a phenolic profile similar to that of tomato fruits [8,10].

**Table 4:** Mass spectral characteristics of phenolics identified in *Solanum indicum* L. berries.

Phenolic acids		
Compound	[M-H]^- ^(m/z)	MS^2^ ions (m/z)
*p*-Coumaric acid	163	119
Caffeic acid	179	135
Coumaroylquinic acids	337	191, 173, 163
Caffeoylquinic acids	353	191, 173, 179
Feruloylquinic acids	367	191, 173, 193
**Flavonoids**		
**Compound**	**[M-H]^- ^(m/z)**	**MS^2^ ions (m/z)**
Naringenin	271	151
Quercetin*	301	151
Quercetin-3- *O*-glucoside	463	301
Kaemferol-glucoside	447	285
Quercetin-3- *O*-rutinoside	609	301
Kaemferol-rutinoside	593	285
Kaemferolrhamnosyl-galactoside*	593	285
Isorhamnetin-rutinoside	623	315

* Identified in red berries of *Solanum indicum *L.

Table 5 shows the content of phenolics of the berries at different maturity stages. Almost all the phenolic acids, but especially coumaroylquinic and caffeoylquinic acids, increased during the ripening progress. Similarly, quercetin-3-*O*-glucoside, quercetin-3-*O*-rutinoside (aka rutin) and kaempferol-3-*O*-rutinoside content of red *Solanum indicum* L. were about ten times higher than that of green and yellow berries, while the aglycone quercetin was identified exclusively in red berries, as well as the isorhamnetin-rutinoside. On the contrary, the content of feruloylquinic and *p*-coumaric acids remained constant during the different ripening stages. 

**Table 5:** Quantification of phenolic compounds in *Solanum indicum* L. berries at different ripening stages.

Phenolic acids						
	*p*-CoA	CA	CoQA	CQA	FQA	
**Green**	18.8 ± 0.7	2.0 ± 1.0	356.0 ± 26.4	932.3 ± 184.8	277.9 ± 11.2	
**Yellow**	19.2 ± 0.9	3.4 ± 0.3	348.4 ± 27.7	887.3 ± 42.4	246.8 ± 7.7	
**Red**	15.8 ± 1.1	18.9 ± 0.8	517.4 ± 34.4	5303.3 ± 523.6	250.2 ± 20.4	
**Flavonoids**						
	NAR	Q-3-Glu	K-Glu	Q-3-Rut	K-Rut	IR-Rut
**Green**	95.5 ± 16.3	38.6 ± 19.3	154.7 ± 8.5	422.4 ± 31.2	378.8 ± 4.5	NQ
**Yellow**	128.2 ± 18.5	36.0 ± 18.0	167.4 ± 13.3	311.3 ± 28.4	363.2 ± 22.4	NQ
**Red**	461.7 ± 71.7	386.8 ± 30.1	800.6 ± 32.3	3820.5 ± 215.1	5869.5 ± 321.2	440.9 ± 15.0

Data expressed as μg/100 g of fresh weight ± SE (n=3); N.Q.: not quantifiable; *p*-CoA: *p*-coumaric acid; CA: caffeic acid; CoQA: coumaroylquinic acids; CQA: caffeoylquinic acids; FQA: feruloylquinic acids; NAR: naringenin; Q-3-Glu: quercetin-3-*O*-glucoside; K-Glu: kaemferol-glucoside ; Q-3-Rut: quercetin-3-*O*-rutinoside; K-Rut: kaemferol-rutinoside; IR-Rut: isorhamnetin-rutinoside.

The trend of phenolic compounds found in *Solanum indicum* L. during ripening was in disagreement with that reported for tomato phenolics, in which chlorogenic and caffeic acids as well as rutin and quercetin gradually decline with maturity progress [10]. However, the flavonoid content of full ripe tomatoes was similar to that of red *Solanum indicum *L. [8]. 

**Table 6:** Pearson’s linear correlation coefficients among *Solanum indicum* L. berries data. ^a^

	FRAP	total phenols	α-carotene	β-carotene	lycopene	ascorbic acid	L*	a*	b*
**FRAP**	1.000								
**polyphenols**	0.068	1.000							
**α-carotene**	0.812**	0.136	1.000						
**β-carotene**	0.853**	0.074	0.937**	1.000					
**lycopene**	0.846**	0.073	0.938**	0.999**	1.000				
**ascorbic acid**	-0.833**	-0.032	-0.908**	-0.966**	-0.966	1.000			
L*	-0.239	0.057	-0.400*	-0.360	-0.381*	0.267	1.000		
a*	0.658**	0.355	0.538**	0.605**	0.585**	-0.537**	0.048	1.000	
b*	-0.017	0.259	-0.196	-0.159	-0.186	0.143	0.852**	0.396*	1.000

^a^ Values followed by * are significant at P < 0.05; values followed by ** are significant at P < 0.01.

### 2.6. Factor Analysis

Data were submitted to factor analysis to identify associations between variables and their ability to discriminate the product at different levels of maturity. Nine variables were used and, among those, only colour values from image analysis were considered as it was proposed as a low-cost method for colour evaluation. Two factors were selected, according to the criteria of an eigenvalue of >1.0. The two factors accounted for 81% of total variance. After computation of a Varimax rotation, it was found that factor 1, with an eigenvalue of 5.20, represents 57.8% of overall variance, while factor 2, with an eigenvalue of 2.09, represents 23.2%. Correlations among variables were also assessed and reported in Table 6.

**Figure 5:**
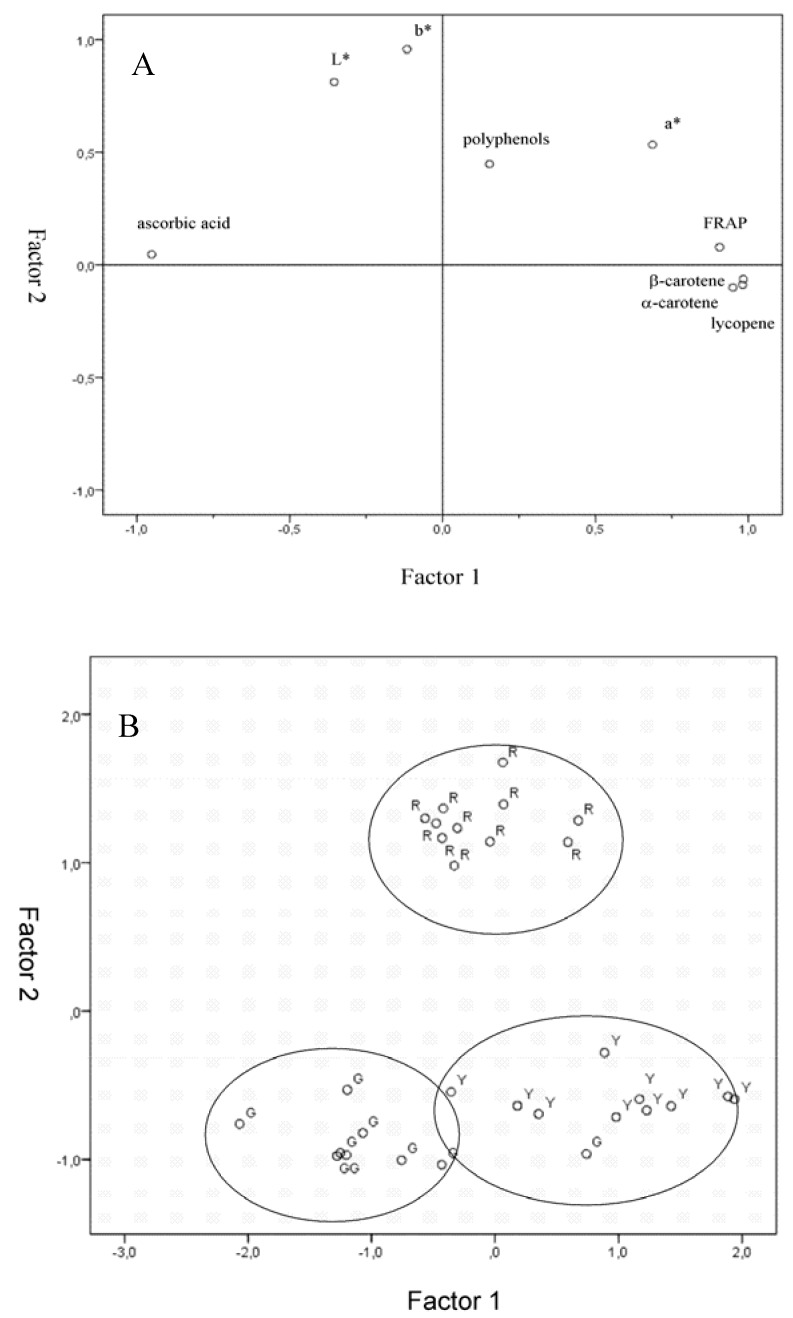
Factor analysis biplots for Gnagnan maturity stage : (A) variables loadings; (B) score loadings.

FRAP values were highly and positively correlated with α-carotene, β-carotene, lycopene and also a* index that, as previously stated, represents the most useful colorimetric index for this kind of product. On the other hand, total polyphenol content was not correlated to any of the parameters considered and FRAP values were inversely correlated to ascorbic acid content.

*Solanum indicum* L. berries factor analysis biplots are reported in Figure 5. Factor 1 was strongly correlated directly to FRAP, α-carotene and β-carotene and lycopene content and inversely to ascorbic acid content. On the other hand, Factor 2 resulted correlated to colorimetric indexes L*, a* and b*. Factorial analysis allowed obtaining a good separation of the different ripening stages (Figure 5B). These results also support for *Solanum indicum* L. berries the common biology-based assessments of *Solanaceae* maturity and quality level, which is largely done by means of inspection systems or by the use of colour only. 

## 3. Experimental

### 3.1. Chemical

Ascorbic acid (A) was purchased from Riedel-de Haën (Sigma-Aldrich, St. Louis, MO, USA). Potassium phosphate monobasic was purchased from Fluka (Sigma-Aldrich, St. Louis, MO, USA). Lycopene, trans-β-carotene, quercetin, rutin, quercetin-3-glucoside, naringenin, caffeic, *p*-coumaric and 3-caffeoylquinic acids, 2,4,6-tripyridyl-*s*-triazine (TPTZ), and 2,6-di-*tert*-butyl-*p*-cresol (BHT) were purchased from Sigma (Sigma-Aldrich, St. Louis, MO, USA). Oxalic acid was purchased from Merck (Darmstadt, Germany). *O*-Phosphoric acid 88% (v/v) was purchased from BDH Chemicals LTD (Poole, England). All chemicals and solvents (all HPLC-grade) were purchased from Carlo Erba Reagents (Milan, Italy) and from Merck (Darmstadt, Germany). High-purity water was produced in the laboratory by using an Alpha-Q system (Millipore, Marlborough, MA, USA).

### 3.2. Solanum indicum *L.* fruits

The *Solanum indicum* L. berries came from Ivory Coast. All the fruits were purchased from a local market, collected at different ripening stages, with different colours (green, yellow and red) and stored at -80 °C prior to analysis. 

### 3.3. Analysis

The analysis of reduced ascorbic acid were performed according to previously described methods [23,24]. The determination of carotenoids was carried out by high-performance liquid chromatography (HPLC) analysis according to Leonardi *et al.* [25]. The phenolic compounds were extracted following the procedure described by Crozier *et al.* [26], and determined by the Folin–Ciocalteu assay [27]. The total antioxidant capacity (TAC) was determined as previously described by Pellegrini *et al.* [20]. Food extracts were immediately analyzed in triplicate for their antioxidant capacity by ferric reducing antioxidant power (FRAP) assay [28]. FRAP values were expressed as millimoles of Fe^2+^ equivalents per 100 g of sample. All extracts were stored at -80 °C prior to analysis.

### 3.4. HPLC-DAD analysis

Both ascorbic acid and carotenoids were analyzed using a Hewlett Packard 1100 separation module equipped with a Waters 2996 Photodiode Array Detector (DAD), using a Waters 717 Plus autosampler, and a Millenium32 data processor (Waters, Milford, MA, USA). The ascorbic acid, lycopene and β-carotene content were quantified using the corresponding standard compound, whereas α-carotene was quantified as β-carotene equivalents.

### 3.5. HPLC-ESI-MS /MS analysis of phenolic compounds

Phenolic compounds were analysed using a Waters 2695 Alliance separation module equipped with a Micromass Quattro Micro API mass spectrometer fitted with an electrospray interface (ESI) (Waters, Milford, MA, USA). Separations were performed using a Waters Atlantis dC18 3 µm (2.1 x 150 mm) reverse phase column (Waters), with the mobile phase, pumped at a flow rate of 0.17 mL/min. We have generated two Multiple Reaction Monitoring (MRM) methods for identification of phenolic acids and flavonoids. For phenolic acid analysis, capillary and cone voltages of 2.8 kV and 30 V, respectively, were used, while the collision energy was set at 20 eV. The analytes were eluted with a 15-min gradient of 5-30% acetonitrile in 1% aqueous formic acid. For flavonoid analysis, capillary and cone voltages were set at 2.8 kV and 35 V, respectively, and the collision energy was 30 eV. Flavonoids were eluted by means of a 10-min gradient of 20-40 % acetonitrile in 1 % aqueous formic acid. 

For all MRM methods, the ESI source worked in negative mode, with a temperature of 120 °C, desolvation temperature of 350 °C, desolvation gas (N_2_) 750 L/h, cone gas (N_2_) 50 L/h, and the collision gas used was argon.

3-*O*-Caffeoylquinic acid, *p*-coumaric acid, caffeic acid, naringenin, quercetin-3-*O*-rutinoside and quercetin-3-*O*-glucoside were all quantified by reference to standard calibration curves. The other cinnamoylquinic acids were quantified in caffeoylquinic acid equivalents by monitoring the loss of cinnamoyl moiety with resulting ionization of quinic acid. The kaempferol-glucoside, on the other hand, was quantified in quercetin-3-*O*-glucoside equivalents by monitoring the loss of sugar moiety with resulting ionization of kaempferol, and flavonol-rutinosides were quantified in quercetin-3-*O*-rutinoside equivalents by monitoring the loss of rutionosyl moiety with resulting ionization of the corresponding aglycone. 

### 3.6. Colour determination

Colour determination was carried out by means of two different methods:

*Colorimeter*: colour determinations were carried out by means of a Minolta reflectance colorimeter (CM 2600d, Minolta Co., Osaka, Japan) equipped with a standard illuminant D65: L* (lightness, black = 0, white = 100), a* (redness > 0, greenness < 0), b* (yellowness, b* > 0, blue < 0) were quantified on each sample using a 2° position of the standard observer. The instrument was calibrated before each analysis with white and black standard tiles. A total of 10 determinations were performed for each sample.

*Image analysis*: samples were scanned by means of a desktop flatbed scanner (Hewlett Packard Scanjet 8200, Palo Alto, CA, USA) at 236 pixels per cm (600 dpi of resolution; true colour – 24 bit), equipped with a cold cathode lamp for reflective scanning. All images were scanned at the same conditions, by positioning on the scanner 10 samples: during image acquisition, the scanner was held in a black box, in order to exclude surrounding light and external reflections. Flatbed scanner colour was characterized and corrected as previously reported by Romani *et al.* [29].

### 3.7. Statistical Analysis

Means and standard deviations (SD) were calculated with SPSS (Version 17.0, SPSS Inc., Chicago, Illinois, USA) statistical software. SPSS was used to verify significant differences between colorimetric and antioxidant data by one-way-analysis of variance (ANOVA) followed by Tukey’s honest significant difference test (HSD) at p ≤ 0.05 to identify differences among groups.

A factor analysis was also performed by means of the same software on the Y-variable data set (physical and nutritional data) to determine if individual variables could be combined to define some underlying multivariate parameter. In factor analysis, linear combinations of the variables are successively computed to maximize overall variability followed by an axis rotation to facilitate interpretation as previously reported by Clèment *et al.* [30]. The first factor explains the highest proportion of data set variability (eigenvalue), the second factor represents the second highest eigenvalue, and so on. Factors are new, independent variables (not correlated among themselves). A value (score) can be calculated for each ripening level on each factor. Factors having an eigenvalue of >1.0 were considered as being of interest for interpretation; they were selected, and a Varimax rotation was done to better distinguish which original variables are most correlated with each factor.

## 4. Conclusions

The present study is the first to fully characterize the antioxidant content and colour of *Solanum indicum* L. berries at different ripening stages. Based on the present results, Gnagnan berries should be consumed at full ripeness in order to benefit from the putatively bioactive molecules present in these *Solanaceae* fruits, such as carotenoids and phenolic compounds. The factorial analysis demonstrated that the ripening degrees of the berries are fully described by FRAP, α-carotene, β-carotene and lycopene content and colorimetric indexes (L*, a* and b*). Moreover, this study demonstrated that by means of colour analysis or even only visual inspection it is possible to choose the best maturity stage of Gnagnan. 

Considering its high content of antioxidants, *Solanum indicum* L. might be considered as an interesting food to improve the antioxidant status of people living in the sub-Saharan Africa, even though further studies are needed in order to understand the impact on the nutritional values of *Solanum indicum* L. of various environmental conditions during the growing period and after harvest.
